# Inferring Genetic Variation and Demographic History of *Michelia yunnanensis* Franch. (Magnoliaceae) from Chloroplast DNA Sequences and Microsatellite Markers

**DOI:** 10.3389/fpls.2017.00583

**Published:** 2017-04-21

**Authors:** Xue Zhang, Shikang Shen, Fuqin Wu, Yuehua Wang

**Affiliations:** ^1^School of Life Sciences, Yunnan UniversityYunnan, China; ^2^Yunnan Research and Monitoring Center of Nature Reserve, Yunnan Institute for Forest Inventory and PlanningKunming, China

**Keywords:** genetic diversity, endemic plant, gene flow, ornamental shrub, microsatellite markers, populations contraction

## Abstract

*Michelia yunnanensis* Franch., is a traditional ornamental, aromatic, and medicinal shrub that endemic to Yunnan Province in southwest China. Although the species has a large distribution pattern and is abundant in Yunnan Province, the populations are dramatically declining because of overexploitation and habitat destruction. Studies on the genetic variation and demography of endemic species are necessary to develop effective conservation and management strategies. To generate such knowledge, we used 3 pairs of universal cpDNA markers and 10 pairs of microsatellite markers to assess the genetic diversity, genetic structure, and demographic history of 7 *M. yunnanensis* populations. We calculated a total of 88 alleles for 10 polymorphic loci and 10 haplotypes for a combined 2,089 bp of cpDNA. *M. yunnanensis* populations showed high genetic diversity (*Ho* = 0.551 for nuclear markers and *Hd* = 0.471 for cpDNA markers) and low genetic differentiation (*F*_*ST*_ = 0.058). Geographical structure was not found among *M. yunnanensis* populations. Genetic distance and geographic distance were not correlated (*P* > 0.05), which indicated that geographic isolation is not the primary cause of the low genetic differentiation of *M. yunnanensis*. Additionally, *M. yunnanensis* populations contracted ~20,000–30,000 years ago, and no recent expansion occurred in current populations. Results indicated that the high genetic diversity of the species and within its populations holds promise for effective genetic resource management and sustainable utilization. Thus, we suggest that the conservation and management of *M. yunnanensis* should address exotic overexploitation and habitat destruction.

## Introduction

Investigating population genetics, which includes genetic diversity, population differentiation, and degree of gene flow, is crucial in the conservation and sustainable utilization of a species (Wuyun et al., 2015). Genetic variation is affected by life cycles, ecological traits, and historical events (Yuan et al., 2008). Thus, studies on plant genetic variation can relate a plant with its evolutionary potential for adaptation and survival under environmental stress and climate change (Wu et al., 2015). For years, population genetics and contributing factors have been investigated with different molecular markers, such as amplified fragment length polymorphism (Landey et al., 2013; Mutegi et al., 2016), inter-simple sequence repeats (Han et al., 2007), and random amplified polymorphic DNA (Ram et al., 2008). Co-dominant nuclear microsatellite markers (nSSRs) are different from dominant markers. nSSRs have desirable advantages for assessing the genetic features of species at individual and population levels, such as locus specificity, high reproducibility, highly polymorphism, and technical simplicity (Kalia et al., 2011; Wambulwa et al., 2016). Chloroplast DNA (cpDNA) is not subjected to recombination and has a low effective population size; thus, it can provide complementary and exact information on population differentiation, genetic diversity level, and demographic history of a species. In addition, cpDNA is uniparentally inherited and thus can inform on gene flow mediated by seed dispersal in angiosperms. Therefore, biparentally inherited microsatellites are often combined with uniparental organelle markers for population genetic studies (Poudel et al., 2014).

*Michelia yunnanensis* Franch. belongs to the genus *Michelia* in the Magnoliaceae family. It is endemic to Yunnan Province, southwest China. The plant is mainly distributed in mountainous thickets, open forests, and forest edges, and is an indispensable subset for evergreen forests in the central and southern parts of Yunnan Province (Qi, 2000). Moreover, *M. yunnanensis* provides economic benefits to local residents given its use in horticultural planting, medicines, and perfume manufacturing (Hao, 1999). Although the species is widely distributed, its natural populations and individuals have dramatically declined in recent years based on our field survey because of excessive anthropogenic collection and habitat destruction.

*M. yunnanensis* has been cultivated and introduced as a horticultural plant because of its graceful appearance, evergreen leaves, aromatic flowers, and strong resistance to wind and toxic gases (Hao, 1999). Its biological characteristics, such as morphological diversity, seed physiology, and tissue culture, have been reported (Han et al., 2010; Song et al., 2013). Detailed studies on the reproductive biology of *M. yunnanensis* are lacking, but the species is believed to be an insect-pollinated outcrosser, similar to other species of the genus *Michellia* (Qi, 2000). Moreover, spice and anti-tumor ingredients (sesquiterpene) have been detected in different parts of the *M. yunnanensis* (Lu and Zhu, 1984; Hu et al., 2010).

Understanding the genetic variation and demographic history of a plant species is essential for conservation decisions and sustainable utilization strategies. The genetic variation and demographic history of wild populations of *M. yunnanensis*, however, have not been studied. In the present study, we combined cpDNA sequences and microsatellite markers to study the genetic diversity and differentiation, genetic structure, and historical demography of *M. yunnanensis*. This study addresses the following questions: what is the degree of genetic diversity and gene flow within/between the species' populations? How does the genetic structure of the current population reflected by the gene flow? Such information is essential for devising optimum strategies regarding conservation priorities for populations and sustainable utilization of this species from a genetic perspective.

## Materials and methods

### Plant materials

**Figure 1:**
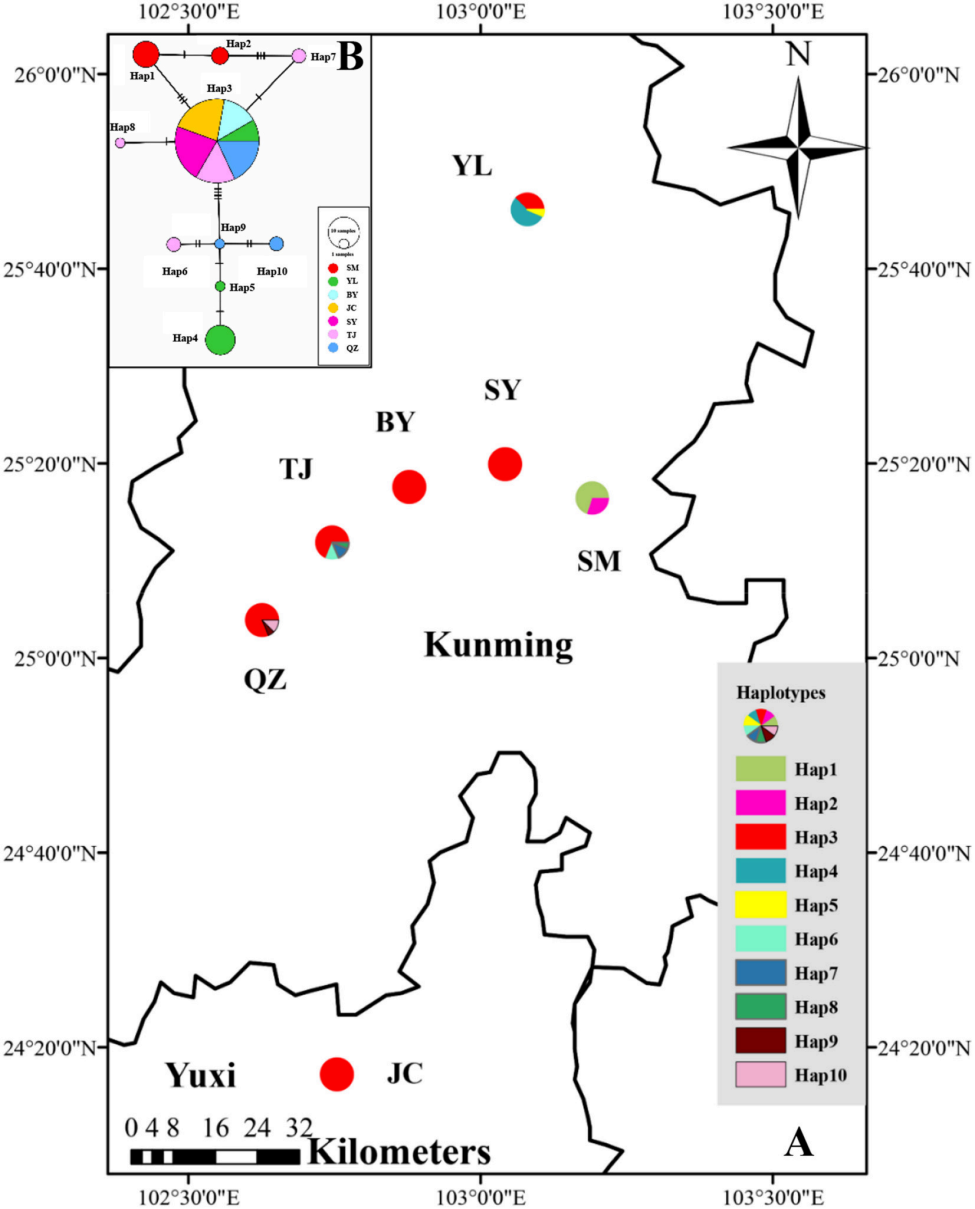
**(A)** Distribution of cpDNA haplotypes detected in 7 populations of *M. yunnanensis*. **(B)** Network of haplotypes of *M. yunnanensis* based on cpDNA. The size of size of the circles corresponds to the frequency of each haplotype, and the vertical molding on branches indicate mutational steps.

Because the populations of this species are still declining due to over-exploitation, 100 samples from 7 natural populations (SM, YL, BY, SY, TJ, QZ, and JC) in Yunnan Province (Table 1) were sampled based on the herbarium specimen records and interviews. Except the population SM and BY, in which only 10 individuals have been found, remaining populations were respectively selected 16 individuals (about 10% of each population, Lu et al., 2002) to study. Further, information on each sampled location and numbers of individuals per population were used for analysis are presented in Figure 1 and Table 1, respectively. To avoid sampling the same genotypes, a minimum distance of 15 m between individuals was maintained (Wu et al., 2015). Young and healthy leaves of every sample were collected and placed immediately in desiccating silica gel, returned to the laboratory, and stored at 4°C for DNA extraction.

**Table 1:** **Details of sample locations, sample size (n), haplotype diversity (***Hd***) and nucleotide diversity (***Pi***) surveyed for cpDNA sequences of ***M. yunnanensis*****.

**Population code**	**Latitude (N°)**	**Longitude (E°)**	**Altitude (m)**	**Individuls (n)**	**Haplotypes (No.)**	**cpDNA**	
						***Hd***	**Pi × 1000**
SM	25.274	103.191	2181	10	Hap1(7) Hap2(3)	0.467	0.220
YL	25.768	103.08	2374	16	Hap3(6) Hap4(9) Hap5(1)	0.575	1.450
BY	25.293	102.878	1714	10	Hap3(10)	0.000	0.000
JC	24.287	102.754	1733	16	Hap3(16)	0.000	0.000
SY	25.332	103.042	1907	16	Hap3(16)	0.000	0.000
TJ	25.097	102.646	1968	16	Hap3(11) Hap6(2) Hap7(2) Hap8(1)	0.525	0.830
QZ	25.065	102.626	2172	16	Hap3(13) Hap9(1) Hap10(2)	0.342	0.850
Total				100		0.471	1.050

*Hd, Haplotype diversity; Pi, Nucleotide diversity*.

### DNA extraction, PCR amplification, and sequencing

Intact genomic DNA was extracted from dried leaves with the modified CTAB method (Doyle, 1991). Extracted DNA was dissolved in 50 μL Tris-EDTA (TE) buffer and stored at −20°C for amplification via polymerase chain reaction (PCR). Through preliminary screening using plastid and nuclear primers, 3 universal cpDNA intergenic spacers (matK, psbA-trnH, and TrnL-trnF) and 10 microsatellite markers were selected from 32 recently developed nuclear microsatellites in Magnoliaceae (Zhao et al., 2009; Sun et al., 2010; Li et al., 2012) on the basis of their clarity and reproducibility.

cpDNA PCR reactions were performed in a total reaction volume of 25 μL that contained 15 ng DNA, 2.5 μL 10 × PCR buffer, 1.5 μL MgCl_2_ (25 mM), 2.0 μL dNTPs (10 mM), 0.5 μL of each primer, 0.4 μL Taq DNA polymerase (5 U/μL; Takara, Shiga, Japan), and 16.6 μL double-distilled water. PCR amplifications were performed in accordance with the following conditions: 3 min of initial denaturation at 94°C; 35 cycles of 1 min at 94°C; 1 min of annealing at 50°, 53°, and 54°C for trnL-trnF, psbA-trnH, and matK, respectively; 1.5 min of extension at 72°C; and 10 min of final extension at 72°C. PCR reactions of 10 microsatellite markers were referenced and modified into a total reaction volume of 20 μL that contained 20 ng DNA, 2.5 μL 10 × PCR buffer, 1.25 μL MgCl_2_ (25 mM), 2.25 μL dNTPs (10 mM), 0.5 μL of each primer, 0.4 μL Taq DNA polymerase (5 U/μL; Takara, Shiga, Japan), and 11.1 μL double-distilled water. For each reaction, we used the following conditions: initial 5 min of denaturation at 94°C, followed by 20 cycles of 50 s at 94°C, 50 s of annealing at Tm under different primers, 1.5 min of extension at 72°C, and a final extension for 7 min at 72°C.

The fluorescent dye (FAM, TAMRA, or HEX) was labeled with the forward SSR primers and visualized on an ABI 3730xl Capillary DNA Analyzer by a professional laboratory (Sangon, Shanghai, China). Fragment sizes were assessed using GeneMapper version 4.0. Meanwhile, all purified PCR products by cpDNA markers were bidirectionally sequenced by Sangon in accordance with standard sequencing protocols.

### Data analysis

#### Data analysis of cpDNA sequence

Sequences were edited and assembled by SeqMan II (Swindell and Plasterer, 1997). Multiple alignments of cpDNA sequences were performed manually with subsequent adjustments by Bioedit version 7.0.4.1 (Hall, 1999). Three cpDNA regions were combined by PAUP^*^ 4.0b10 (Swofford, 2002). These regions showed a significant rate of homogeneity (*P* > 0.5) under a congruency test. The combined cpDNA sequences were used in the following analyses.

We calculated haplotypes and variable sites from aligned cpDNA sequences using DnaSP version 5.0 (Librado and Rozas, 2009). We tested genetic diversity within and among populations by calculating *Nei* nucleotide diversity (Pi) and haplotype diversity (*Hd*) indices. Using ArcGis version 10.2 (http://desktop.arcgis.com), the haplotypes of combined cpDNA sequences were plotted on the map of the region from which the samples were collected. Within-population gene diversity (*H*_*S*_) and gene diversity in total populations (*H*_*T*_) were calculated using Permut version 1.0 (http://www6.bordeaux-aquitaine.inra.fr/biogeco/Production-scientifique/Logiciels/Contrib-Permut/Permut). Meanwhile, the level of population differentiation (*G*_*ST*_) and phylogenetically ordered alleles (*N*_*ST*_) were also calculated by this software to test for the presence of phylogeographic structures (Pons and Petit, 1996).

Arlequin version 3.11 (Excoffier et al., 2005) was used to analyze molecular variance (AMOVA, Excoffier et al., 1992) and estimate the hierarchical genetic structure assigned within and among populations. The signification of the estimated *F*_*ST*_ across populations was tested with 10^3^ permutations.

Popart (http://popart.otago.ac.nz/examplenex.shtml) was used to construct a genealogical haplotype network to estimate the degree of relatedness per haplotype. Indels were treated as single mutational events. Phylogenetic relationships of cpDNA haplotypes in *M. yunnanensis* populations were inferred without an outgroup by Bayesian methods in MrBayes version 3.1.2 (Ronquist and Huelsenbeck, 2003) and neighbor-joining method in Mega version 6.06 (Tamura et al., 2013).

Evolutionary rates estimated for *M. yunnanensis* were converted to 1.01 × 10^−9^ (Graur and Li, 2000) with BEAST version 1.6.1 (Drummond and Rambaut, 2007). Markov Chain Monte Carlo (MCMC) analysis was run for 10^7^ iterations with a burn-in of 10^6^ under the HKY model and a strict clock. The most suitable model (HKY) was determined by Modeltest in Mega version 6.06 (Tamura et al., 2013). MCMC samples were inspected in TRACER version 1.5 (Rambaut and Drummond, 2004). All runs were coalesced to the stationary distribution with an adequate sampling (effective sample size > 200 after the burn-in). The results were visualized in FigTree version 1.4.2 (http://tree.bio.ed.ac.uk/software/tracer/).

To further investigate population expansion and signatures of demographic change in populations, we used DnaSP version 5.0 (Librado and Rozas, 2009) and Arlequin version 3.11 (Excoffier et al., 2005) to calculate pairwise mismatch distribution and neutrality tests (Tajima's *D* and Fu's *F*_*S*_, Tajima, 1989; Fu, 1997). The sum of squared deviations (SSD) between the observed and expected mismatch distributions and raggedness index were computed by Arlequin version 3.11 (Excoffier et al., 2005). *P*-values were also calculated by this program.

#### Data analysis of microsatellite markers

Dataset editing and formatting were performed in GenAlEx version 6.3 (Peakall and Smouse, 2006). To assess the genetic diversity of each locus and each population, we used GenAlEx version 6.3 (Peakall and Smouse, 2006) to calculate descriptive statistics, such as the number of alleles (*Na*), effective number of alleles (*N*_*E*_), number of private alleles (*N*_*P*_), expected heterozygosity (*He*), observed heterozygosity (*H*_*O*_), information index (*I*), percentage of polymorphic loci (*PPB*), and fixation index (*F*_*IS* =_ 1-Ho/He) across all microsatellite loci. We mutually corrected statistics with FSTAT version 2.9.3 (Goudet, 1995) and POPGENE version 1.31 (Yeh et al., 1997). Total genetic diversity for species (*H*_*T*_), and coefficient of gene differentiation (*G*_*ST*_) were estimated using FSTAT version 2.9.3 (Goudet, 1995), and the rarefied allelic richness (*Ra*) was also calculated by this program according to Leberg (2002). Linkage disequilibrium (LD) was also investigated at the 5% statistical significance level among loci pairs with 1,000 permutations using this software and then corrected by the sequential Bonferroni method (Rice, 1989). Hardy-Weinberg equilibrium (HWE) was tested for each locus and each population using Genepop version 4.1.4 (Yeh et al., 1997). Arlequin version 3.11 (Excoffier et al., 2005) was used to compute the differentiation between pairwise populations with *F*_*ST*_, as well as to estimate historical gene flow between population pairs using Wright's principles *Nm* = (1 − *F*_*ST*_)/4*F*_*ST*_ (Wright, 1931). In addition, the ratio (r) of pollen to seed gene flow was estimated according to Ennos' (1994) formula: *r* = [(1/*F*_*ST*(*B*)_ − 1) − 2 (1/*F*_*ST*(*M*)_ − 1)]/(1/*F*_*ST*(*M*)_ − 1), in which *F*_*ST*(*B*)_ and *F*_*ST*(*M*)_ were F statistics for nSSRs and cpDNA markers calculating by Arlequin respectively.

By performing Mantel tests, isolation by distance (IBD) was tested by analyzing the correlation of *F*_*ST*_/(1 − *F*_*ST*_) with geographic distance for all population pairs using GenAlEx version 6.3 (Peakall and Smouse, 2006). The genetic structures of sampled populations were estimated through an unweighted pair group mean analysis (UPGMA) using TFPGA version 1.3 (Miller, 1997) with 5,000 permutations. Principal coordinates analysis (PCA) was visualized R software by *ape* package to assess genetic relationship at individual levels (Paradis et al., 2004). An admixture model-based cluster method was implemented with SSR data using STRUCTURE version 2.2 (Pritchard et al., 2000). Models were tested for *K*-values of 1–7 with 20 independent runs per set and a burn-in of 1 × 10^5^ iterations and 1 × 10^5^ subsequent MCMC steps. The second-order rate of change of the log probability, with respect to the number of clusters (Δ*K*), was used as an additional estimator of the likeliest number of genetic clusters (Evanno et al., 2005). The optimal number of clusters was selected by plotting LnP(D) values against Δ*K* values in STRUCTURE HARVESTER version 0.6.8 (Earl, 2012).

To test for recent bottleneck events, we used BOTTLENECK version 1.2.02 (Piry et al., 1999). We explored the demographic history of populations using the stepwise mutation (SMM) and two-phased (TPM) models and a heterozygosity excess test. The computation was performed under two methods (Sign and Wilcoxon tests), which were applied as the numbers of population <20. Moreover, we computed the Garza-Williamsion index (GWI) using Arlequin version 3.11 (Excoffier et al., 2005). GWI detects genetic bottlenecks that lasted for several generations prior to the rapid demographic recovery of the population (Garza and Williamson, 2001; Williamson-Natesan, 2005). When 7 or more loci were analyzed, GWI was lower than the critical *Mc* value of 0.68, which indicated that the populations decreased in population size or vice versa (Garza and Williamson, 2001; Excoffier et al., 2005).

## Result

### cpDNA sequences

The combined cpDNA sequences had a 2,089-bp consensus length. The consensus sequence contained the cpDNA fragments matk, psbA-trnH, and trnL-trnF, which are 842, 429, and 818 bp in length, respectively (GenBank accession numbers: KY400136-KY400147). A total of 17 variable sites were identified from the cpDNA dataset, containing 13 SNPs, 3 indels, and 14 segregating sites. Finally, 10 haplotypes (Hap1-10) were obtained from 7 populations of *M. yunnanensis* (Table 1 and Figure 1A). Except for Hap3, which was the most abundant haplotype with a wide distribution in 6 populations, other fixed haplotypes were unique in each population. The values of haplotype diversity (*Hd*) and nucleotide diversity per site (*Pi*) in total populations were 0.471 and 1.06, respectively (Table 1). The highest values (*Hd* = 0.575, *Pi* = 1.45) occurred in population YL, followed by TJ (0.525, 0.83), and SM (0.467, 0.22); whereas no diversity was found in the 3 remaining populations (BY, JC, and SY). Total genetic diversity (*H*_*T*_ = 0.522) was higher than the average intrapopulation diversity (*H*_*s*_ = 0.243). The permutation test revealed a higher value for *N*_*ST*_ (0.728) than *G*_*ST*_ (0.534) in *M. yunnanensis* populations. The difference between the two statistics, however, was not significant (*P* > 0.05). This result indicated the absence of phylogenetic structure among populations. Moreover, the AMOVA result indicated significant genetic differentiation among all populations (*F*_*ST*_ = 0.636, *P* < 0.01) with most chloroplast diversity occurring among populations (63.56% of the total variance) compared with only 36.44% partitioned within populations (Table 2).

**Table 2:** **Analysis of molecular variance (AMOVA) based on cpDNA and nSSR for populations of ***M. yunnanensis*****.

**Primer**	**Source of variation**	**d.f**.	**Sum of squares**	**Variance components**	**Percentage of Variation(%)**	***F_*ST*_***
cpDNA	Among Pops	6	84.755	0.956Va	63.560	0.636^***^
	within Pops	93	50.975	0.548Vb	36.440	
	Total	99	135.730	1.504		
nSSR	Among Pops	6	44.402	0.165Va	5.760	0.058^**^
	within Pops	93	522.263	2.706Vb	94.240	
	Total	199	556.665	2.871		

*** , P < 0.001, most significant difference;

** *, 0.05 < p < 0.15, significant difference*.

**Figure 2:**
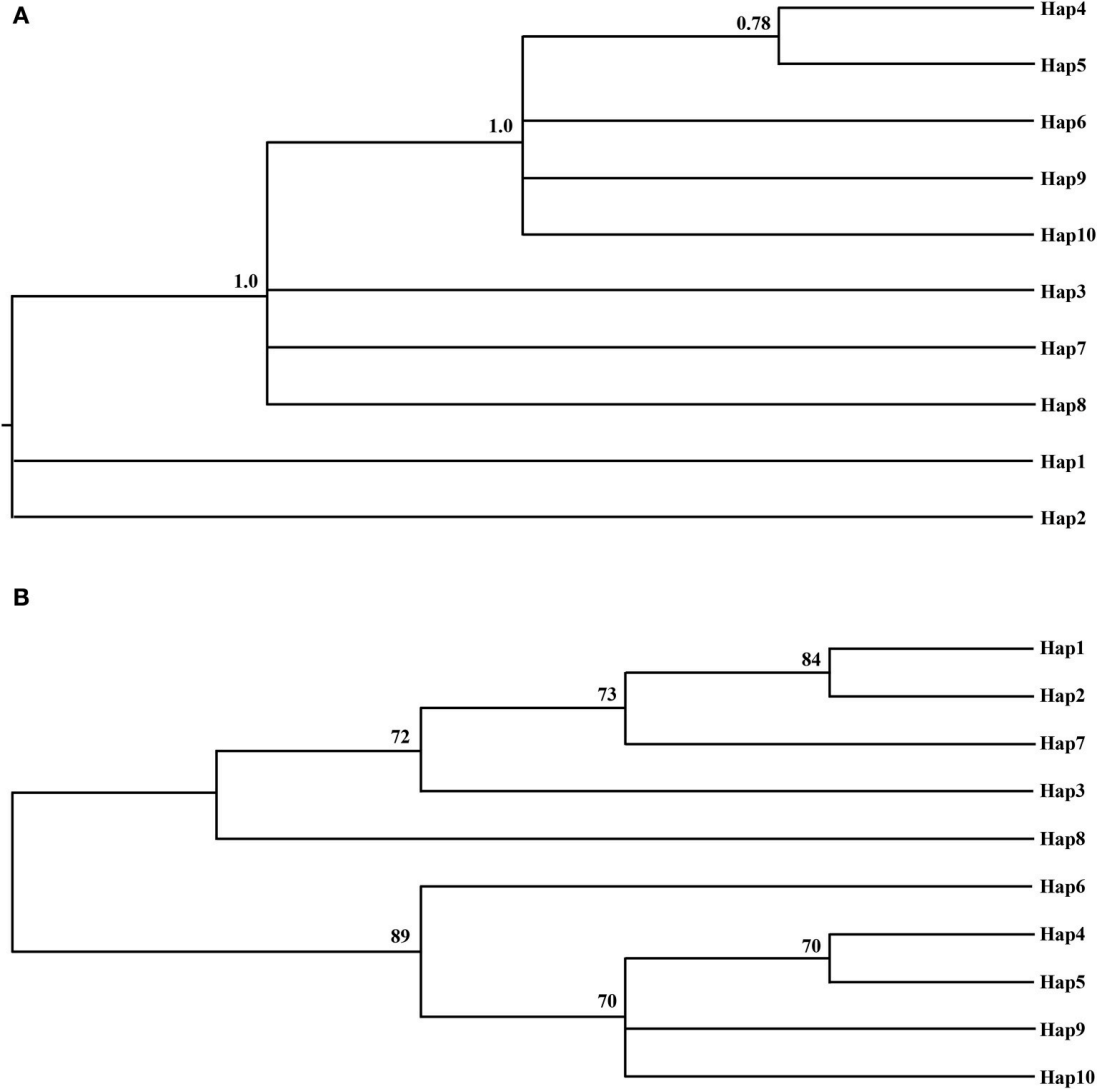
**The Bayesian tree (A)** and the Neighbor-joning consensus tree (NJ) **(B)** based on haplotypes of combined cpDNA sequences. The numbers on branches indicate the posterior probability and bootstraps values, respectively.

The phylogenetic relationships among the haplotypes, which were obtained via neighbor-joining and Bayesian methods, revealed that 10 cpDNA haplotypes clustered into 2 clades (Figure 2). The neighbor-joining consensus tree was further clarified to show that Hap 4 was closely related to Hap 5, Hap 3 was more closely related to Hap 7 than Hap 8, and Hap 9 was more closely related to Hap 10 than Hap 6 (Figure 2B). The results of the genealogical haplotype network diagram supported the congruent phylogenetic relationships of the cpDNA haplotypes, which showed that Hap 3 and Hap 9 were distributed in the central position and that the remaining haplotypes were distributed in the outside nodes of the reticulate evolutionary diagram (Figure 1B). This distribution pattern predicted that Hap 3 and Hap 9 likely represent the ancestral haplotypes for this geographic area. Hap 3, which was the most abundant haplotype, was widely distributed in most populations.

The neutrality test showed that between Fu and Li'*D* values, Fu and Li'*F* were positive, whereas the values of Tajima's *D* and Fu'*Fs* were negative but non-significant (Supplementary Table S1). These results indicated the low probability of recent population expansion in *M. yunnanensis*. Moreover, the mismatch distribution analysis, which used a multimodal graph (Supplementary Figure 1A) indicated no recent population expansion and had a SSD of 0.058 (*P* = 0.083) and a HRag of 0.204 (*P* = 0.241; Supplementary Table S1).

The Bayesian skyline plot showed the demographic scenario of the species. *M. yunnanensis* maintained a constant population size for a long time until ~20,000–30,000 years ago when a contraction event occurred (Supplementary Figure 1B).

### SSR data

A total of 88 alleles were identified in 10 polymorphic loci, ranging from 3 (ssr22) to 22 (ssr27) among 7 *M. yunnanensis* populations with an average of 8.8 alleles per locus (Supplementary Table S2). As presented in Supplementary Table S3, except for 3 (ssr14, 22, 27, *P* < 0.001) loci, almost all the remaining loci conform to the equilibrium state in HWE test (*P* > 0.05). Five of the 45 locus pairwise showed LD (*P* < 0.05) after Bonferroni correction, but not significant. Diversity estimates from 10 microsatellites varied among populations (Table 3). The total number of alleles (*N*_*T*_) of each population varied from 38 (SM) to 46 (QZ); rarefied allelic richness (*Ra*) varied from 3.539 (YL) to 4.175 (BY); number of private alleles (*N*_*P*_) varied from 2 (SM) to 9 (QZS); and percentage of polymorphic loci (PPB) varied from 90% (BY, TJ, SY, QZS) to 100% (SM, YL, JC). The lowest values of observed heterozygosity (*H*_*O*_ = 0.513) and expected heterozygosity (*H*_*E*_ = 0.513) all occurred in population SY, whereas the maximum values of observed heterozygosity (*H*_*O*_ = 0.605) and expected heterozygosity (*H*_*E*_ = 0.581) were distributed in YL and TJ populations, respectively. The above parameters showed that *M. yunnanensis* populations have a relatively high genetic diversity.

**Table 3:** **Genetic diversity of populations in ***M. yunnanensis*****.

**Pop**	***Na***	***Ne***	***I***	***Ho***	***H_*E*_***	***F_*IS*_***	***N_*T*_***	***Np***	***Ra***	**PPB (%)**
SM	3.800	2.675	0.951	0.590	0.516	−0.093 (*ns*)	38	2	3.687	100.0
YL	4.100	2.471	1.005	0.605	0.560	−0.047 (*ns*)	41	6	3.539	100.0
BY	4.300	2.870	1.023	0.556	0.520	−0.016 (*ns*)	43	5	4.175	90.0
JC	4.800	2.955	1.065	0.544	0.547	0.038 (*ns*)	48	6	4.009	100.0
TJ	4.500	3.312	1.114	0.556	0.581	0.075 (*ns*)	45	4	4.033	90.0
SY	4.200	2.666	0.989	0.513	0.513	0.034 (*ns*)	42	2	3.729	90.0
QZ	4.600	2.645	1.027	0.517	0.525	0.047 (*ns*)	46	9	3.904	90.0
Mean	4.329	2.799	1.025	0.554	0.537	0.038	43.286	4.857	3.868	94.3

Na, The mean number of alleles; Ne, No. of Effective Alleles; I, Shannon's Information Index; H_O_, Observed Heterozygosity; He, Expected Heterozygosity; N_T_, No. of Total Alleles; N_P_, No. of Private Alleles; Ra: rarefied allelic richness; F_IS_, Fixation Index; PPB(%), Percentage of Polymorphic Loci; ns, no significant difference; ^***^, P < 0.001, most significant difference; ^**^, p < 0.01, most significant difference; ^*^, p < 0.05, significant difference; ns, no-significance.

**Figure 3:**
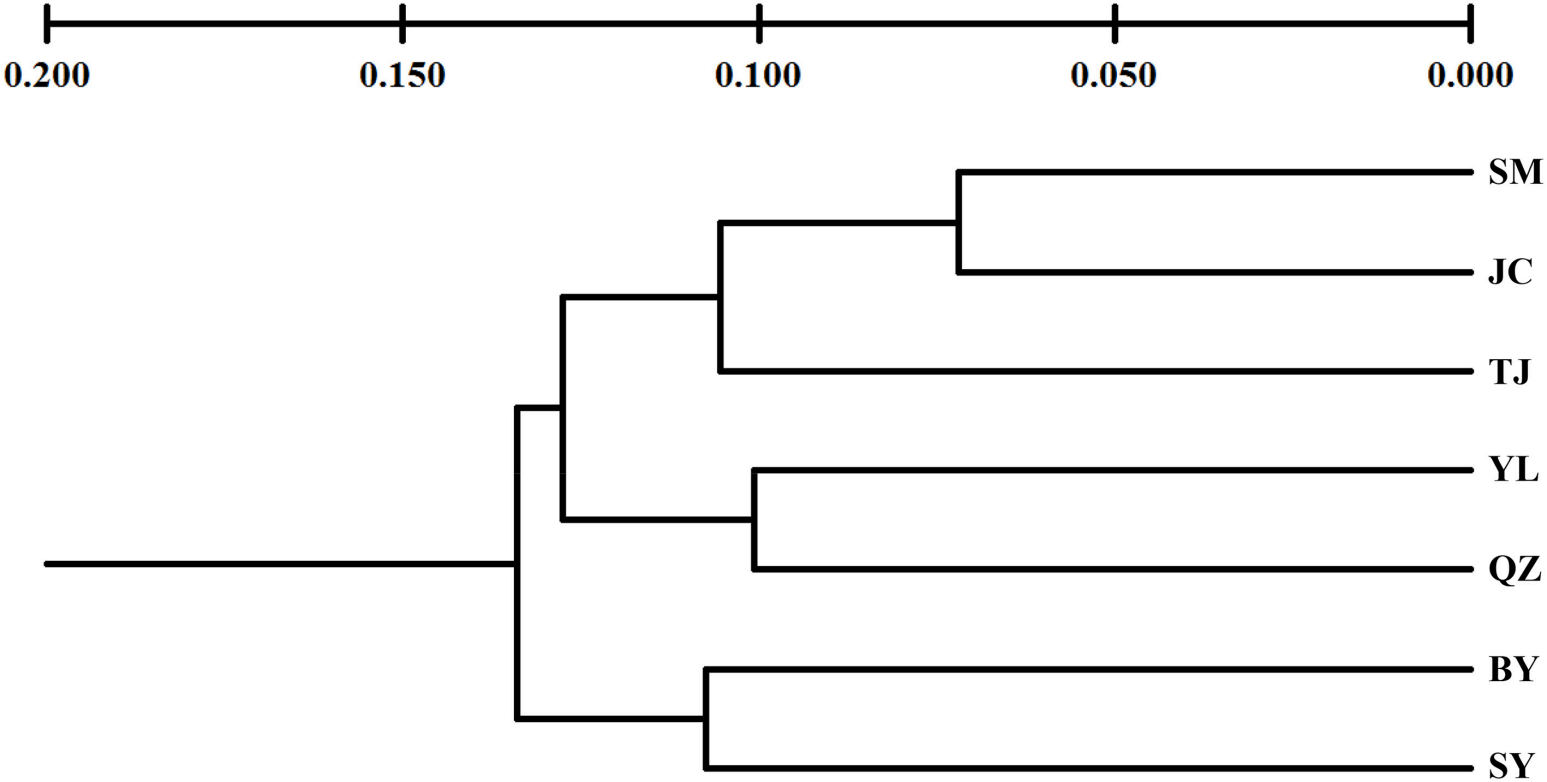
**An unweighted pair-group method with arithmetic averages (UPGMA) phenogram of 7 populations of ***M. yunnanensis***, based on microsatellite markers**.

**Figure 4:**
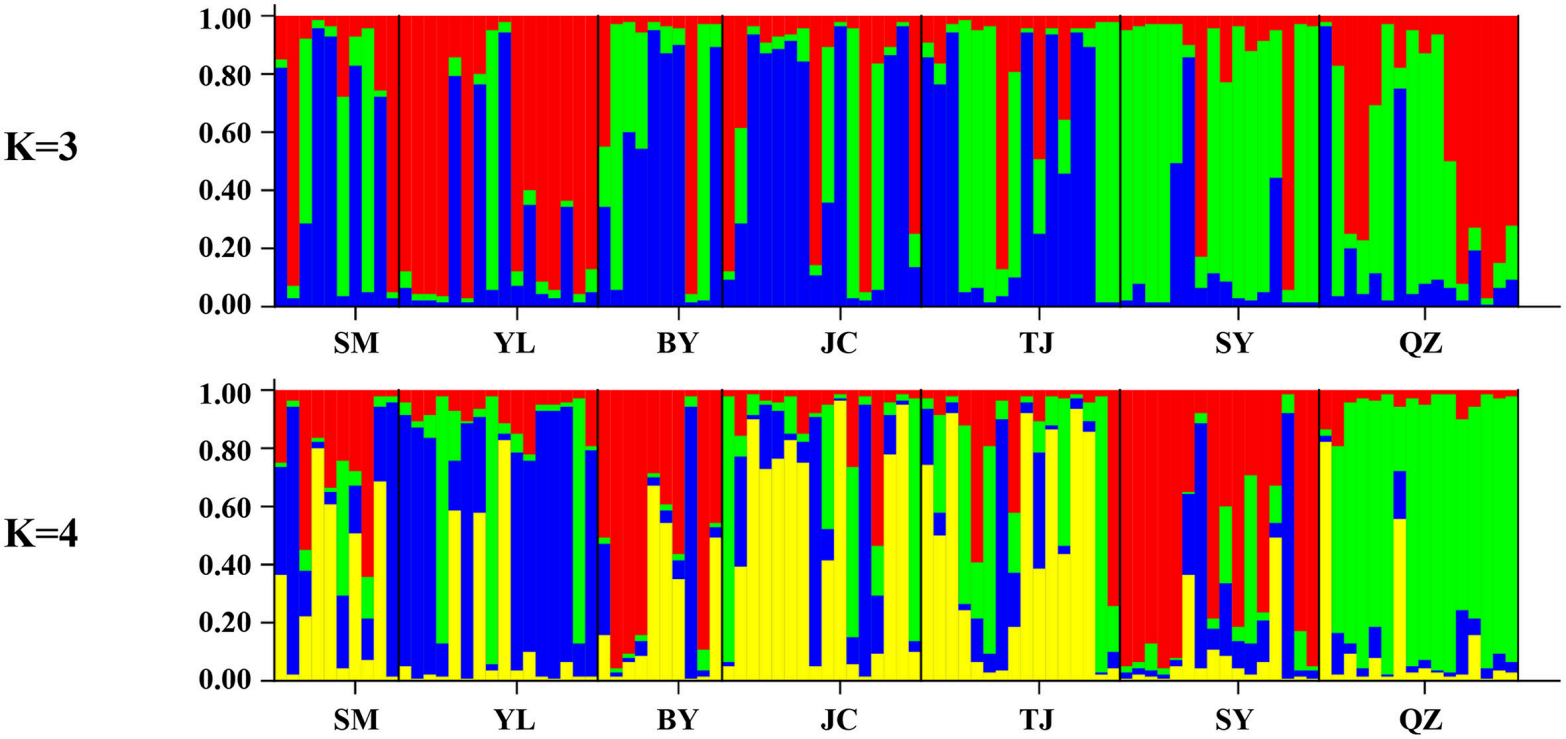
**Bayesian inference of number of clusters (***K*** = 3 and ***K*** = 4) for ***M. yunnanensis*****.

Unlike the cpDNA sequences analysis results, AMOVA analysis of SSR data showed that 94.24 and 5.76% of genetic variation were partitioned in and among populations, respectively (Table 2). Evidence of inbreeding was not found in any population because we did not find any significant fixation index values (*F*_*IS*_, Supplementary Table S2, Table 3). A low genetic differentiation was observed among populations (*F*_*ST*_ = 0.058). Estimates of gene flow between each pair of the 7 populations are shown in Table 4. The maximum gene flow was generated from SM and JC populations (14.184), whereas the minimum gene flow was generated from TJ and JC populations (0.660). The ratio of pollen to seed gene flow was 26.3791. The dendrogram obtained with the UPGMA clustering method showed that the 7 *M. yunnanensis* populations were separated into two clusters with high bootstrap values (100) (Figure 3). Similarly, PCA grouped natural populations into two clusters at the individual level but failed to cluster individuals at the population level (Supplementary Figure 2). STUCTURE analysis, which used the Δ*K* method, showed that the optimal *K*-value was 4 for Δ*K* = 3.362 and the second fit value was 3 for Δ*K* = 2.712, although a higher degree of admixture might be noticed. However, the results either from *K* = 4 or *K* = 3 both failed to differentiate populations (Figure 4). The correlation between genetic and geographic distances, as determined by mantel tests, were not significant (*P* = 0.10) and indicated no significant effect of IBD (Supplementary Figure 3).

**Table 4:** **The gene flow between 7 populations of ***M. yunnanensis*****.

**Pop**	**SM**	**YL**	**BY**	**JC**	**TJ**	**SY**	**QZS**
SM	0.000						
YL	5.314	0.000					
BY	9.600	3.786	0.000				
JC	14.184	4.024	4.337	0.000			
TJ	4.447	3.606	2.925	0.660	0.000		
SY	3.434	3.573	4.792	3.323	3.628	0.000	
QZ	2.913	5.166	2.180	3.788	4.660	4.393	0.000

Bottleneck analysis showed that the allele distribution per population was a normal L-shaped distribution under TPM and SMM, which conformed to the mutation-drift equilibrium. Non-significant probabilities of Wilcoxon and Sign tests (*P* > 0.05) indicated that all populations did not experience a recent bottleneck event (Table 5). However, all the GWI (Table 5) of 7 populations were lower than the critical Mc value of 0.68, which indicated that *M. yunnanensis* populations underwent a historical demographic reduction.

**Table 5:** **Bottleneck analysis for 7 populations of ***M. yunnanensis*****.

**Pop**	**T.P.M**		**S.M.M**		**Mode shift**	**Garza-Williamson index**
	**Sign test (ns)**	**Wilcoxon test (ns)**	**Sign test (ns)**	**Wilcoxon test (ns)**		
SM	0.367	0.625	0.178	0.695	L	0.394
YL	0.621	0.625	0.372	0.695	L	0.331
BY	0.4112	0.625	0.352	0.846	L	0.310
JC	0.363	0.193	0.369	0.625	L	0.412
TJ	0.351	0.275	0.062	0.557	L	0.378
SY	0.331	0.492	0.599	0.922	L	0.296
QZ	0.204	0.770	0.065	0.193	L	0.293

*P is test for heterozigosity excess, ns, no significant difference*.

## Discussion

### Genetic diversity of *M. yunnanensis*

In general, rare or endemic species with small populations always have low levels of genetic diversity (Poudel et al., 2014; Yoichi and Tomaru, 2014). However, previous studies have reported high levels of genetic diversity in some small population species (Wu et al., 2015; Meng et al., 2016). The present study revealed that *M. yunnanensis* is characterized by relatively higher genetic diversity than other related species, such as *M. coriacea* (Zhao et al., 2012), *M. maudiae* (Sun et al., 2011), and *M. formosana* (Lu et al., 2002). Notably, the values of within-population genetic diversity (*He* = 0.588) in *M. yunnanensis* were considerably higher than the values of “narrow” species (*He* = 0.420) and approached those of “widespread” species (*He* = 0.620, Nybom, 2004). Life span, reproductive mode, and breeding system are the most important factors in genetic diversity (Nybom, 2004; Feng et al., 2014; Wu et al., 2015). Outcrossing species tend to have higher genetic diversity and heterozygosity than self-pollinated ones, and massive gene flows mitigate the loss of genetic diversity through genetic drift (Nybom, 2004). The present study detected high gene flow for *M. yunnanensis*, which has an outcross breeding system (Qi, 2000). Moreover, negative or close to zero *F*_*IS*_ values proved the high outcrossing rate of the species. The genetic variation of a species reflects its long-term evolution and adaptation to climate changes (Feng et al., 2014): a remnant perennial usually maintains higher levels of intrapopulation genetic diversity than short-lived species (Nybom, 2004). Thus, the high genetic diversity observed in *M. yunnanensis* may be attributed to its long-lived habit.

### Genetic differentiation and structure in *M. yunnanensis*

In *M. yunnanensis*, 5.76% of genetic variation was partitioned among populations for SSR markers, whereas 63.56% genetic variation was partitioned among populations for cpDNA. We preliminarily speculated that this discordance is usually related to the different markers and a high level of effective gene flow. In general, the comparative analysis between biparentally and maternally inherited microsatellite markers provides complementary, but often contrasting information on genetic structure and differentiation because of their different inheritance modes and evolutionary rates (Birky et al., 1989). Han et al. (2015) also proposed that cpDNA variation reflects a past change in population demographics, whereas microsatellite variation infers recent events in the population. Thus, we inferred low genetic differentiation among current populations of *M. yunnanensis*. This result supported the conclusion that range-restricted or endemic species generally exhibit lower differentiation among populations (Nybom and Bartish, 2000).

Genetic structure is determined by demography and gene flow, as well as the scale and temporal stability of the environment used by a given species to complete its life cycle (Côté et al., 2013). A topoclimate with long-term stability provides a favorable environment for plants and pollinators (Gugger et al., 2013). In the present study, the relative contribution of pollen to seed gene flow was 26.3791, which indicated that pollen dispersal played an extremely important role in weakening the genetic structure of *M. yunnanensis*. Furthermore, the widely distributed haplotype (Hap 3) deeply decreased the level of differentiation among populations. Therefore, we speculated that substantial gene flow likely caused the low genetic differentiation and indistinct phylogeographic structure of *M. yunnanensis* (Allendorf, 1983).

Population assignment cannot provide an optimal phylogeographic structure when difference between populations is low (Sun et al., 2011). We found that the correlation between genetic and geographic distances was non-significant among *M. yunnanensis* populations (*P* > 0.05), although the evolutionary trees and genealogical haplotype network showed two reciprocally monophyletic lineages with robust support. However, based on a meta-analysis of isolation by distance, Jenkins et al. (2010) proposed that IBD studies included more populations were more likely to obtain a significant correlation between species' genetic and geographic distances. In addition, the sample size can have a significant effect on the estimation of expected heterozygosity nSSR markers (Nybom, 2004; Poudel et al., 2014). Although previous studies suggest that 20 to 30 individuals should be sampled in microstallite studies, they were also proposed that genetic diversity is prerequisite for research on endangered or endemic populations even if sample sizes are less than ideal (Hale et al., 2012; Pruett and Winker, 2008). Therefore, considering the relative small sample size in the present study, we suggest that the species' genetic characteristics requires further investigation by expanding the sampling scale.

### Demographic history of the *M. yunnanensis* populations

Bottleneck analysis indicated that no recent bottleneck event occurred in *M. yunnanensis*. However, the Bayesian skyline plot for cpDNA showed that the populations significantly decreased ~20,000–30,000 years ago. The result is supported by the microsatellite-based GWI (GWI < MC). Thus, we speculated that Quaternary glaciers (20,000–30,000 years ago) may have affected the genetic variations and structure of this long-lived species (Hewitt, 2000; Gugger et al., 2013). *M. yunnanensis* responded to glacial and interglacial influences in different ways, such as shifting the latitude or elevation of their ranges, population expansion, and population contraction (Davis and Shaw, 2001). As a remnant plant from the last glacial period, the current geographical distribution of *M. yunnanensis* may be related to climate oscillation (Hewitt, 2000; Gong et al., 2015), which formed the foundation of current genetic variations in *M. yunnanensis* (Qiao et al., 2010).

The neutrality test and mismatch distribution indicated that no recent population expansion occurred in this species. *M. yunnanensis* is different from some surviving glacial plants, such as *Tetracentron sinense* (Sun et al., 2014) and *Taxus wallichiana* (Gao et al., 2007), but is similar to *Ginkgo biloba* (Gong et al., 2008) and *Cathaya argyrophylla* (Wang and Ge, 2006). These differences and similarities likely resulted from the propagation system and seed dispersal capability of *M. yunnanensis* (Cheng et al., 2015). Seed dispersal is limited by the plant's large, heavy seeds. Moreover, the species' seed dispersal vectors have decreased because of habitat damage (Han et al., 2010). Beyond limitations on the seed itself, however, most seeds suffer negative effects from human disturbance during dispersal and collection by local residents.

## Conclusion

Information on the genetic diversity, genetic structure, and demographic history of endemic species can aid the development of appropriate conservation and management strategies. In the present study, the genetic variation and demographic history of seven wild *M. yunnanensis* populations were first investigated with microsatellite markers and cpDNA sequences. The plant showed high levels of genetic diversity and low levels of genetic differentiation among populations. Given that a high number of private haplotypes and high microsatellite diversity, populations SM, YL, TJ, QZ should be considered as distinct evolutionary significant units (ESUs). These populations with a wider genetic base may be significant to adapt future scenarios and used as source material for germplasm banks and/or nurseries (Poudel et al., 2014). In addition, species with low levels of genetic differentiation will not suffer major reductions in genetic diversity upon the loss of a population (Dodd and Helenurm, 2002), the conservation and management of *M. yunnanensis* should focus on exotic overexploitation and habitat destruction. Little evidence of inbreeding and high gene flow was detected. Reduced gene flow may be expected, however, given that the species is undergoing dramatic population decline and isolation.

Through the comprehensive analysis of demographic history, we observed that *M. yunnanensis* has undergone a past population contraction ~20,000–30,000 years ago. This event was likely influenced by Quaternary glaciations. Furthermore, no recent population expansion occurred in this species because of their limited seed dispersal over long distances and human interference. Therefore, we suggest establishing protection zones or plots in the distribution areas of *M. yunnanensis*, controlling seed collection and minimizing the habitat destruction by local people. Moreover, we advise relevant local departments to develop corresponding conservation actions and mitigation measures to utilize *M. yunnanensis*, as well as to raise conservation awareness and implement rational management in distribution areas.

## Author contributions

SS, XZ, and YW initiated and designed the research. SS obtained funding for this study. XZ, FW, and SS collected the materials and performed the experiments, SS and XZ analyzed the data and wrote the paper. All authors read and approved the manuscript.

## Funding

This study was financially supported by grant 31560224 and 31360155 from the National Natural Science Foundation of China.

### Conflict of interest statement

The authors declare that the research was conducted in the absence of any commercial or financial relationships that could be construed as a potential conflict of interest.
