# Supplementary material for: Inferring Genetic Variation and Demographic History of Michelia yunnanensis Franch. (Magnoliaceae) from Chloroplast DNA Sequences and Microsatellite Markers
Source: Front Plant Sci. 2017 Apr 21;8:583. doi: 10.3389/fpls.2017.00583 (PMC5399939; doi:10.3389/fpls.2017.00583)
Supplement: Supplementary file 1 [file Table1.DOC]

Supplementary Material

**Inferring genetic variation and demographic history of *Michelia yunnanensis* Franch. (Magnoliaceae) from chloroplast DNA sequences and microsatellite markers**

**Authors:** Xue Zhang, Shen Shikang*,

***Address for Correspondence:** Shen Shikang, School of Life Sciences, Yunnan University, No. 2 Green lake North road Kunming, Yunnan, 650091, the People’s Republic of China. Telephone:+86-871-65031412; Fax:+86-871-65031412;

**E-mail:** yunda123456@126.com

***Supplementary Table 1*** *Parameters of neutrality tests and mismatch analysis based on cpDNA of M. yunnanensis*

| **Marker** | **Tajima' *D*** | **Fu and Li' *D**** | **Fu and Li' *F**** | **Fu' *Fs*** | **SSD** | **raggedness** |
| --- | --- | --- | --- | --- | --- | --- |
| cpDNA | -0.513 | 1.549 | 0.957 | -0.415 | 0.058 | 0.204 |
