# Supplementary material for: Inferring Genetic Variation and Demographic History of Michelia yunnanensis Franch. (Magnoliaceae) from Chloroplast DNA Sequences and Microsatellite Markers
Source: Front Plant Sci. 2017 Apr 21;8:583. doi: 10.3389/fpls.2017.00583 (PMC5399939; doi:10.3389/fpls.2017.00583)
Supplement: Supplementary file 2 [file Table2.DOC]

Supplementary Material

**Inferring genetic variation and demographic history of *Michelia yunnanensis* Franch. (Magnoliaceae) from chloroplast DNA sequences and microsatellite markers**

**Authors:** Xue Zhang, Shen Shikang*,

***Address for Correspondence:** Shen Shikang, School of Life Sciences, Yunnan University, No. 2 Green lake North road Kunming, Yunnan, 650091, the People’s Republic of China. Telephone:+86-871-65031412; Fax:+86-871-65031412;

**E-mail:** yunda123456@126.com

**Supplementary Table 2** Genetic diversity of 10 microsatellite loci for the 7 populations in *M. yunnanensis*

| **Locus** | ***A_R_*** | ***N_A_*** | ***A_E_*** | ***I*** | ***H_O_*** | ***H_E_*** | ***F_IS_*** | **Hs** | **H_T_** | ***G_ST_*** |
| --- | --- | --- | --- | --- | --- | --- | --- | --- | --- | --- |
| ssr1 | 4.441 | 6 | 2.617 | 1.264 | 0.561 | 0.621 | -0.049 | 0.545 | 0.600 | 0.091 |
| ssr9 | 4.320 | 9 | 2.945 | 1.331 | 0.840 | 0.664 | -0.358 | 0.643 | 0.662 | 0.029 |
| ssr10 | 4.192 | 10 | 2.840 | 1.295 | 0.844 | 0.651 | -0.356 | 0.647 | 0.651 | 0.006 |
| ssr13 | 2.451 | 6 | 1.682 | 0.701 | 0.516 | 0.408 | -0.362 | 0.378 | 0.400 | 0.054 |
| ssr14 | 8.355 | 14 | 8.315 | 2.283 | 0.510 | 0.884 | 0.304 | 0.807 | 0.890 | 0.093 |
| ssr16 | 3.344 | 4 | 2.511 | 1.050 | 1.000 | 0.605 | -0.660 | 0.610 | 0.606 | -0.008 |
| ssr22 | 2.090 | 3 | 1.857 | 0.677 | 0.010 | 0.464 | 0.978 | 0.445 | 0.477 | 0.066 |
| ssr24 | 2.007 | 5 | 1.153 | 0.323 | 0.070 | 0.134 | 0.359 | 0.112 | 0.125 | 0.106 |
| ssr27 | 10.728 | 22 | 13.060 | 2.756 | 0.535 | 0.928 | 0.347 | 0.871 | 0.931 | 0.064 |
| ssr30 | 3.442 | 9 | 2.080 | 1.018 | 0.639 | 0.522 | -0.251 | 0.522 | 0.524 | 0.005 |
| Mean |  | 8.8 | 3.906 | 1.270 | 0.551 | 0.588 | -0.005 | 0.558 | 0.587 | 0.051 |
| Total |  | 88 |  |  |  |  |  |  |  |  |

*Note: N_A_, No. of Alleles; A_E_, No. of Effective Alleles; I, Shannon's Information Index; H_O_, Observed Heterozygosity; H_E_, Expected Heterozygosity; A_R_, Allelic richness; F_IS_, Fixation Index; H_S_, gene diversity within populations; H_T_, total diversity for species; G_ST_, Coefficient of gene differentiation.*
