# Supplementary material for: Inferring Genetic Variation and Demographic History of Michelia yunnanensis Franch. (Magnoliaceae) from Chloroplast DNA Sequences and Microsatellite Markers
Source: Front Plant Sci. 2017 Apr 21;8:583. doi: 10.3389/fpls.2017.00583 (PMC5399939; doi:10.3389/fpls.2017.00583)
Supplement: Supplementary file 3 [file Table3.DOC]

Supplementary Material

**Inferring genetic variation and demographic history of *Michelia yunnanensis* Franch. (Magnoliaceae) from chloroplast DNA sequences and microsatellite markers**

**Authors:** Xue Zhang, Shen Shikang*,

***Address for Correspondence:** Shen Shikang, School of Life Sciences, Yunnan University, No. 2 Green lake North road Kunming, Yunnan, 650091, the People’s Republic of China. Telephone:+86-871-65031412; Fax:+86-871-65031412;

**E-mail:** yunda123456@126.com

**Supplementary Table 3** *P*-value of Hardy-Weinberg equilibrium test for 7 populations of *M. yunnanensis*

| **Pop** | **ssr1** | **ssr9** | **ssr10** | **ssr13** | **ssr14** | **ssr16** | **ssr22** | **ssr24** | **ssr27** | **ssr30** | **All loci** |
| --- | --- | --- | --- | --- | --- | --- | --- | --- | --- | --- | --- |
| SM | 1.000ns | 0.339ns | 0.990ns | 1.000ns | 0.076ns | 1.000ns | 0.001** | / | 0.002** | 1.000ns | 0.069ns |
| YL | 0.931ns | 0.554ns | 1.000ns | 1.000ns | 0.003** | 1.000ns | 0.000*** | 0.061ns | 0.011* | 1.000ns | 0.074ns |
| BY | / | 1.000ns | 1.000ns | 1.000ns | 0.378ns | 1.000ns | 0.001** | / | 0.004* | 0.377ns | 0.155ns |
| JC | 0.635ns | 1.000ns | 1.000ns | 1.000ns | 0.000*** | 1.000ns | 0.000*** | 0.097ns | 0.000*** | 1.000ns | 0.003** |
| TJ | 0.412ns | 1.000ns | 0.036* | 0.999ns | 0.002** | 1.000ns | 0.000*** | / | 0.000*** | 0.931ns | 0.000*** |
| SY | 0.001** | 0.656ns | 0.091ns | 1.000ns | 0.000*** | 1.000ns | 0.003** | / | 0.055ns | 1.000ns | 0.000*** |
| QZS | 0.503ns | 0.042* | 0.013* | 1.000ns | 0.509ns | 1.000ns | 0.003** | / | 0.034* | 0.007** | 0.000*** |
| All Pop | 0.151ns | 0.958ns | 0.392ns | 1.000ns | 0.000*** | 1.000ns | 0.000*** | 0.026* | 0.000*** | 0.683ns | 0.000*** |

*Note: ***, P<0.001, most significant difference; **, p<0.01, most significant difference; *, p<0.05, significant difference; ns,no-significance; /, Monomorphic.*
