# Supplementary material for: Inferring Genetic Variation and Demographic History of Michelia yunnanensis Franch. (Magnoliaceae) from Chloroplast DNA Sequences and Microsatellite Markers
Source: Front Plant Sci. 2017 Apr 21;8:583. doi: 10.3389/fpls.2017.00583 (PMC5399939; doi:10.3389/fpls.2017.00583)

Supplementary Material

Inferring genetic variation and demographic history of *Michelia yunnanensis* Franch. (Magnoliaceae) from chloroplast DNA sequences and microsatellite markers

Authors: Xue Zhang, Shen Shikang*,

***Address for Correspondence:** Shen Shikang, School of Life Sciences, Yunnan University, No. 2 Green lake North road Kunming, Yunnan, 650091, the People's Republic of China. Telephone:+86-871-65031412; Fax:+86-871-65031412;

E-mail: yunda123456@126.com

Supplementary Figure 2 Principal coordinates analysis (PCA) from 7 populations of 100 individuals of *M. yunnanensis*

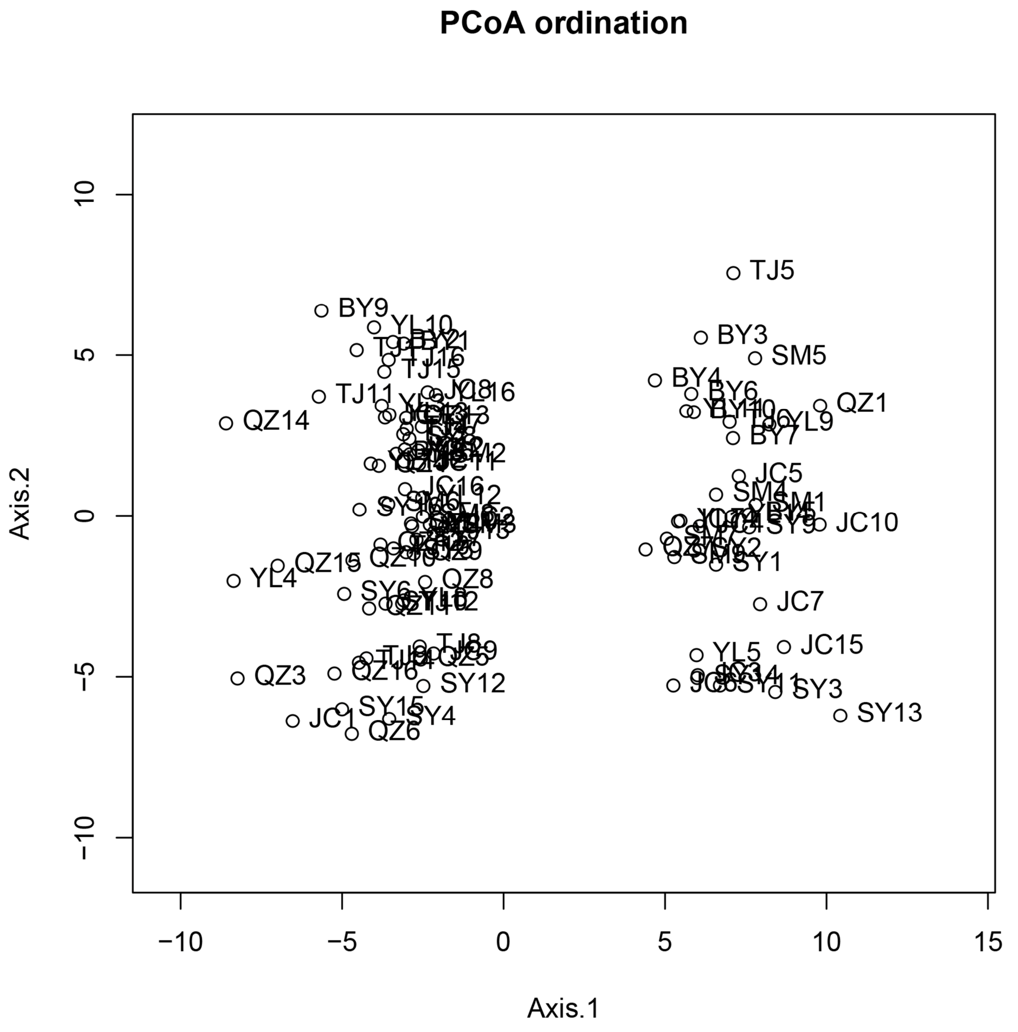

Supplement: Supplementary file 5 [file Image2.PDF]
